# Association of Stroke and Myocardial Infarction Symptom Knowledge with Cancer Screening Participation Among Patients with Hypertension and Diabetes: A Cross-Sectional Study in Korea

**DOI:** 10.3390/healthcare14172782

**Published:** 2026-09-01

**Authors:** Min Soo Kim, Mina Suh, Hye-Sun Lee, Jae Kwan Jun, Hyeon Ji Lee

**Affiliations:** 1 National Cancer Control Institute, National Cancer Center, Goyang 10408, Republic of Korea; soo931108@gmail.com (M.S.K.);; 2 Department of Public Health, General Graduate School of Dankook University, Cheonan 31116, Republic of Korea; 3 Digital Contents School, Hallym University, Chuncheon 24252, Republic of Korea

**Keywords:** hypertension, diabetes, myocardial infarction, stroke, health knowledge, cancer screening

## Abstract

**Background/Objectives:** Patients with hypertension or diabetes often share common risk factors for both cardiovascular diseases and cancer. Although health knowledge is associated with preventive health behaviors, the association between knowledge of stroke and myocardial infarction (MI) symptoms and cancer screening participation remains poorly understood. **Methods:** This cross-sectional study analyzed data from the 2023 Community Health Survey, including 45,949 adults with hypertension and 20,357 with diabetes aged ≥40 years. Multivariate logistic regression was used to examine the association between knowledge of stroke and MI symptoms and cancer screening participation. Subgroup analyses were conducted according to age and sex. **Results:** Among patients with hypertension, knowledge of stroke and MI symptoms was significantly associated with higher odds of undergoing cancer screening. Similar associations were observed among patients with diabetes for stroke and MI symptom knowledge. Subgroup analysis revealed that the association for stroke knowledge was significantly more pronounced among adults aged 40–59 years with hypertension, while remaining age and sex subgroup variations were exploratory. **Conclusions:** Knowledge of stroke and MI symptoms was associated with participation in cancer screening among adults with hypertension and diabetes. These findings suggest that knowledge of cardiovascular symptoms may reflect overall engagement in preventive health behaviors, including cancer screening.

## Simple Summary

Patients with hypertension or diabetes are at increased risk of developing cancer, yet participation in routine cancer screening is often deprioritized owing to the management of these chronic conditions. This study examined whether knowledge of the warning signs of stroke and myocardial infarction (MI) is associated with cancer screening participation among these patients. Analysis of data from the 2023 Community Health Survey showed that individuals with greater awareness of cardiovascular and cerebrovascular symptoms were significantly more likely to undergo cancer screening. Particularly among patients with hypertension, the association for stroke knowledge was significantly more pronounced in middle-aged adults (40–59 years), while other subgroup variations were exploratory. Overall, these findings suggest that cardiovascular symptom knowledge may reflect broader preventive health engagement and inform future health education strategies.

## 1. Introduction

Advances in medical technology have increased life expectancy; however, chronic diseases, including cardiovascular disease, cancer, and diabetes, remain leading causes of death [1]. As the burden of chronic diseases continues to grow, early detection and preventive interventions have become increasingly important in public health [2]. Cancer screening improves survival, enhances quality of life, and reduces treatment costs [3,4,5]. In Korea, the National Health Insurance Service (NHIS) provides free or low-cost cancer screening at recommended intervals ranging from 6 months to 2 years, depending on the cancer type [6], contributing to a steady increase in screening participation [7]. However, participation remains suboptimal among vulnerable populations, posing an ongoing public health challenge.

Myocardial infarction (MI) and stroke are life-threatening conditions that often present with nonspecific or subtle early symptoms, making timely diagnosis challenging [8]. Awareness of their warning signs enables individuals to recognize potential symptoms and seek prompt medical care, thereby reducing the risk of severe complications [9,10]. Stroke, MI, and cancer share several major risk factors—including hypertension, diabetes, and obesity [11,12]. Emerging evidence suggests a biological link between atherosclerosis (the precursor to MI) and carcinogenesis through shared pathways of oxidative stress and systemic inflammation [13].

Patients with hypertension and diabetes share multiple systemic risk factors for cancer, making participation in population-based cancer screening programs a public health priority [11,12,14,15]. Previous international and domestic studies have extensively demonstrated that disease-specific knowledge improves medication adherence and lifestyle modifications for that specific condition [16,17,18]. However, a significant research gap remains regarding whether awareness of acute, life-threatening cardiovascular symptoms translates into broader, proactive preventive actions across different disease categories, such as cancer screening. Furthermore, frequent healthcare visits among individuals with hypertension or diabetes increase opportunities to receive opportunistic recommendations from healthcare professionals [19]. Consequently, it remains unclear whether higher participation in cancer screening reflects an individual’s intrinsic health literacy or is simply a byproduct of more frequent healthcare utilization. This study provides an incremental contribution to the literature by bridging this gap, utilizing a large, nationally representative dataset to evaluate whether acute symptom knowledge serves as an independent proxy for comprehensive preventive health behaviors.

To construct a standardized logical research framework, we integrated Andersen’s Behavioral Model of Health Services Use [20], which posits that healthcare utilization is driven by predisposing, enabling, and need factors. Within this framework, specific health knowledge (e.g., stroke and MI symptom awareness) functions as a predisposing factor reflecting health literacy, which may facilitate the uptake of preventive services. Based on this model, our primary hypothesis was that patients with hypertension and diabetes who recognize stroke and MI warning signs are more likely to participate in routine cancer screening. Furthermore, given the established domestic and international variations in health behaviors, we pre-specified hypotheses for subgroup analyses: we hypothesized that the strength of this association would vary by sex (due to differing health information-seeking behaviors) and age (reflecting age-specific perceptions of distinct disease threats). Accordingly, the objective was to elucidate the association between knowledge of acute cardiovascular symptoms and cancer screening participation, thereby providing a public health rationale for integrating cardiovascular education into comprehensive preventive care.

## 2. Materials and Methods

### 2.1. Study Design, Data Collection, and Recruitment of Participants

This cross-sectional study conducted a secondary analysis of data from the 2023 Community Health Survey (CHS III). The CHS is a legally mandated survey that supports the development and evaluation of regional healthcare plans and generates comparable regional health statistics for adults aged ≥19 years [21]. Since 2008, the Korea Disease Control and Prevention Agency has conducted the survey annually at the county (si/gun/gu) level using a complex, stratified, multistage probability sampling design to ensure national representativeness. Each annual survey includes approximately 900 participants per region (totaling around 200,000 participants nationwide). Data were accessed on 24 November 2024, for research purposes. No information identifying individual participants was available during or after data collection.

### 2.2. Sample Size Calculation, Inclusion, and Exclusion Criteria

**Figure 1:**
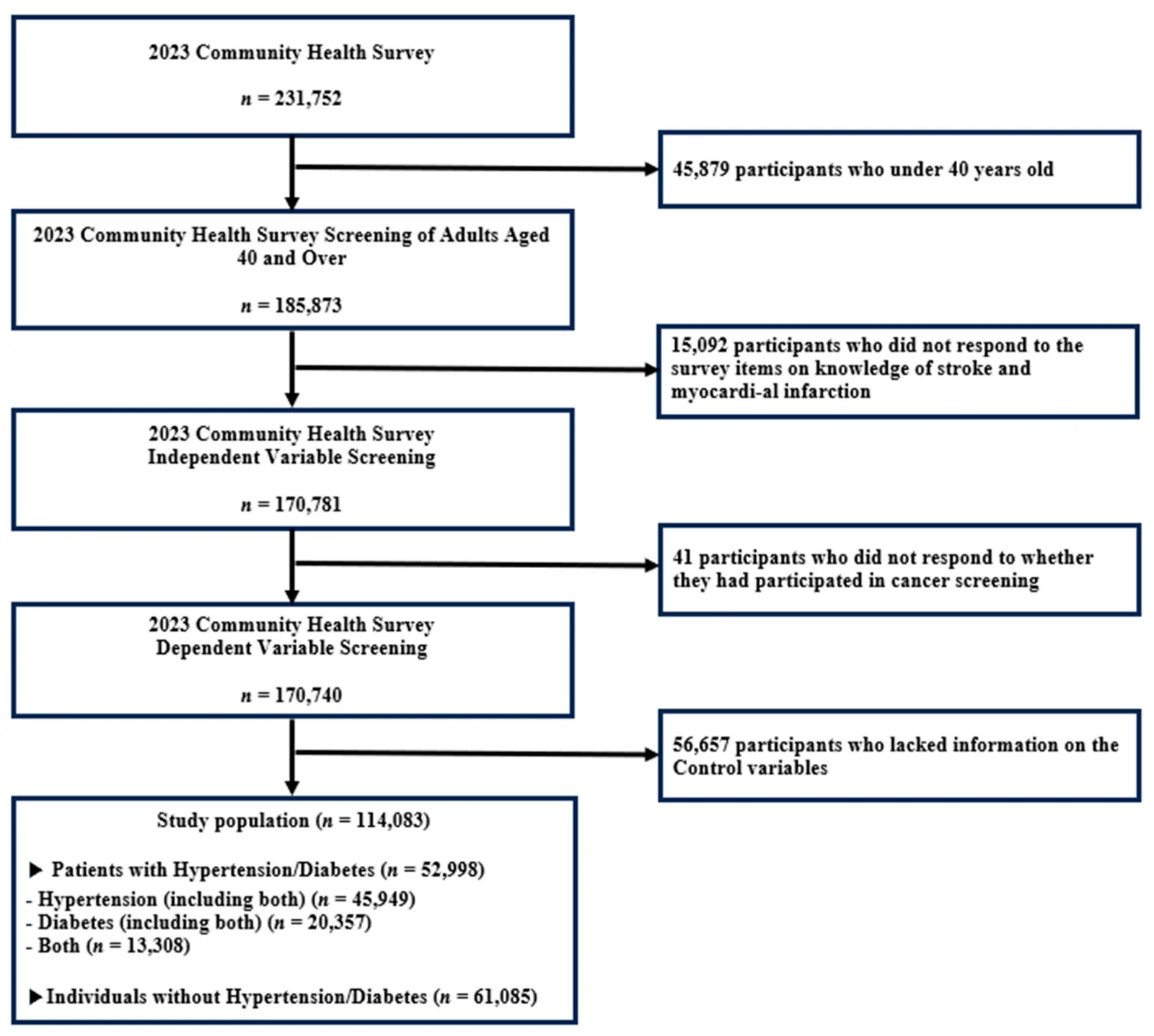
Flow diagram of study population selection from the 2023 Community Health Survey.

As this study utilized a pre-existing national dataset, a formal a priori sample size calculation enrollment was not applicable; however, the large national sample provided sufficient statistical power for the analyses. Among the 231,752 respondents to the 2023 CHS, 185,873 adults aged ≥40 years met the initial inclusion criterion. Conversely, individuals with (1) missing responses to questions assessing knowledge of stroke and MI symptoms (*n* = 15,092), (2) missing responses regarding cancer screening participation (*n* = 41), and (3) missing data for study covariates (*n* = 56,657) were excluded (total excluded, *n* = 71,790; Figure 1). The final analytical sample comprised 114,083 participants. Of these, 52,998 had hypertension/diabetes and constituted the primary study population. This group included 45,949 participants with hypertension, 20,357 with diabetes, and 13,308 with both conditions. The remaining 61,085 participants without either condition served as the comparison group. Potential selection bias resulting from participant exclusions was evaluated by comparing the sociodemographic characteristics of included and excluded participants (Table S1).

### 2.3. Criteria of Diagnosis and Laboratory Data

As the CHS is a nationwide interview-based epidemiological survey rather than a clinical registry, clinical laboratory data (e.g., blood glucose and lipid profiles) and physical measurements were unavailable. Accordingly, hypertension and diabetes were defined based on participants’ self-reported history of a physician diagnosis of each condition.

### 2.4. Independent Variables

The independent variables were knowledge of stroke and MI symptoms. The assessment instrument has demonstrated robust reliability and validity and has been widely used in nationwide epidemiological studies [22,23,24]. In the present study sample, the internal reliability (Cronbach’s α) was 0.84 for the stroke symptom scale and 0.82 for the MI symptom scale. Knowledge of stroke was assessed using the five warning signs: unilateral weakness of the face, arm, or leg; slurred speech or difficulty understanding speech; sudden vision loss in one eye or double vision; dizziness or loss of balance; and sudden severe headache. Responses were scored as 1 for “yes” and 0 for “no.” Similarly, knowledge of MI was assessed using five symptoms: sudden pain or discomfort in the jaw, neck, or back; sudden weakness, dizziness, nausea, or cold sweats; sudden chest pain, pressure, or squeezing; sudden pain or discomfort in the arm or shoulder; and sudden shortness of breath. Responses were scored using the same method. The total score ranged from 0 to 5 points, with higher scores indicating greater knowledge of stroke and MI. Knowledge was analyzed using two approaches. In the primary analysis, participants scoring 5 were classified as the “aware group,” whereas those scoring 0–4 points were classified as the “unaware” group. This strict dichotomization aligns with previous epidemiological studies [9,25,26] and was designed to capture comprehensive symptom recognition while minimizing the effect of partial guessing. Recognizing all major warning signs is considered a critical threshold for appropriate and timely health-seeking behavior in acute settings.

### 2.5. Dependent Variables

The dependent variable was participation in cancer screening, assessed using the survey question: “In the past 2 years, have you undergone a cancer screening to assess your health, despite having no particular health issues?” Responses were recorded as “yes” or “no.” The 2-year assessment period aligns with the screening intervals recommended by the South Korean National Cancer Screening Program (NCSP) for all major target cancers, including stomach (2 years), colorectal (annual fecal occult blood test), liver (6 months), breast (2 years), cervical (2 years), and lung (2 years) [6]. By contrast, several Western guidelines recommend longer screening intervals for certain cancers [27,28]. Therefore, the 2-year timeframe serves as a reasonable survey indicator of recent screening participation within the context of the Korean NCSP while minimizing misclassification.

### 2.6. Control Variables

To align with the conceptual framework, covariates were systematically selected based on Andersen’s model. While symptom knowledge served as the primary predisposing variable of interest, sociodemographic predisposing covariates included sex, age, and educational level. Enabling factors comprised marital status, residential area, employment status, and average monthly household income. Need factors included self-rated health status, smoking status, alcohol consumption, regular physical activity, obesity (body mass index ≥ 25 kg/m^2^) [29], and depressive symptoms. Unmet healthcare needs were included to account for potential differences in healthcare access and utilization.

### 2.7. Analytical Approach and Statistics

To correctly account for the complex, stratified, multistage probability sampling design of the CHS, all variance estimations and regression analyses incorporated the survey strata, primary sampling units (clusters), and sampling weights. Specifically, chi-square tests comparing the distribution of cancer screening participation were performed using the PROC SURVEYFREQ procedure. Multivariable logistic regression, adjusted for covariates, was conducted using the PROC SURVEYLOGISTIC procedure to examine the association between knowledge of stroke and MI symptoms and participation in cancer screening. As a sensitivity analysis, knowledge scores were additionally modeled as continuous variables (0–5) to evaluate graded associations. Potential multicollinearity between stroke and MI symptom knowledge was assessed using the variance inflation factor (VIF). All VIF values were <2.5, indicating no evidence of multicollinearity. Notably, age and sex are important modifiers of both cardiovascular disease risk and cancer screening guidelines [30,31,32,33]. As national screening recommendations and healthcare utilization patterns vary by these factors, stratified subgroup analyses were conducted by sex and age (<60 and ≥60 years). Additionally, robustness analyses were performed among individuals without hypertension or diabetes and those with both conditions to determine whether the observed associations were restricted to specific patient groups. Finally, to address potential overadjustment bias arising from the inclusion of unmet healthcare needs, which may function as a mediator rather than a true confounder, an additional sensitivity analysis was conducted by excluding this variable from the multivariable models. Associations were expressed as odds ratios (ORs), confidence intervals (CIs), and *p*-values. Significance was defined as a *p*-value of <0.05. All statistical analyses were performed using the SAS statistical software package (version 9.4; SAS Institute Inc., Cary, NC, USA).

## 3. Results

### 3.1. Sample Characteristics

A total of 114,083 participants were included in the final analysis (Figure 1). Baseline sociodemographic characteristics of the included participants and excluded individuals (*n* = 71,790) are provided in Table S1. Given the large sample size, all compared variables exhibited significant differences (*p* < 0.001). Notably, most participants were excluded due to missing data on average monthly household income (*n* = 52,994). The excluded participants were more likely to be employed (67.0%) compared with the included participants (49.8%). The study population comprised 52,998 participants with hypertension/diabetes, including 45,949 with hypertension, 20,357 with diabetes, and 13,308 with both conditions. An additional 61,085 individuals without either condition served as the comparison group.

Table 1 summarizes the sociodemographic characteristics of the study participants by chronic disease status. Across all groups, most participants were aged ≥60 years, and the distributions of educational attainment and household income were consistent with those of the Korean population with chronic diseases. Among participants with hypertension or diabetes, those with knowledge of stroke and MI symptoms were more likely to undergo cancer screening than those without such knowledge.

### 3.2. Association Between Knowledge of Stroke and Myocardial Infarction and Participation in Cancer Screening

**Figure 2:**
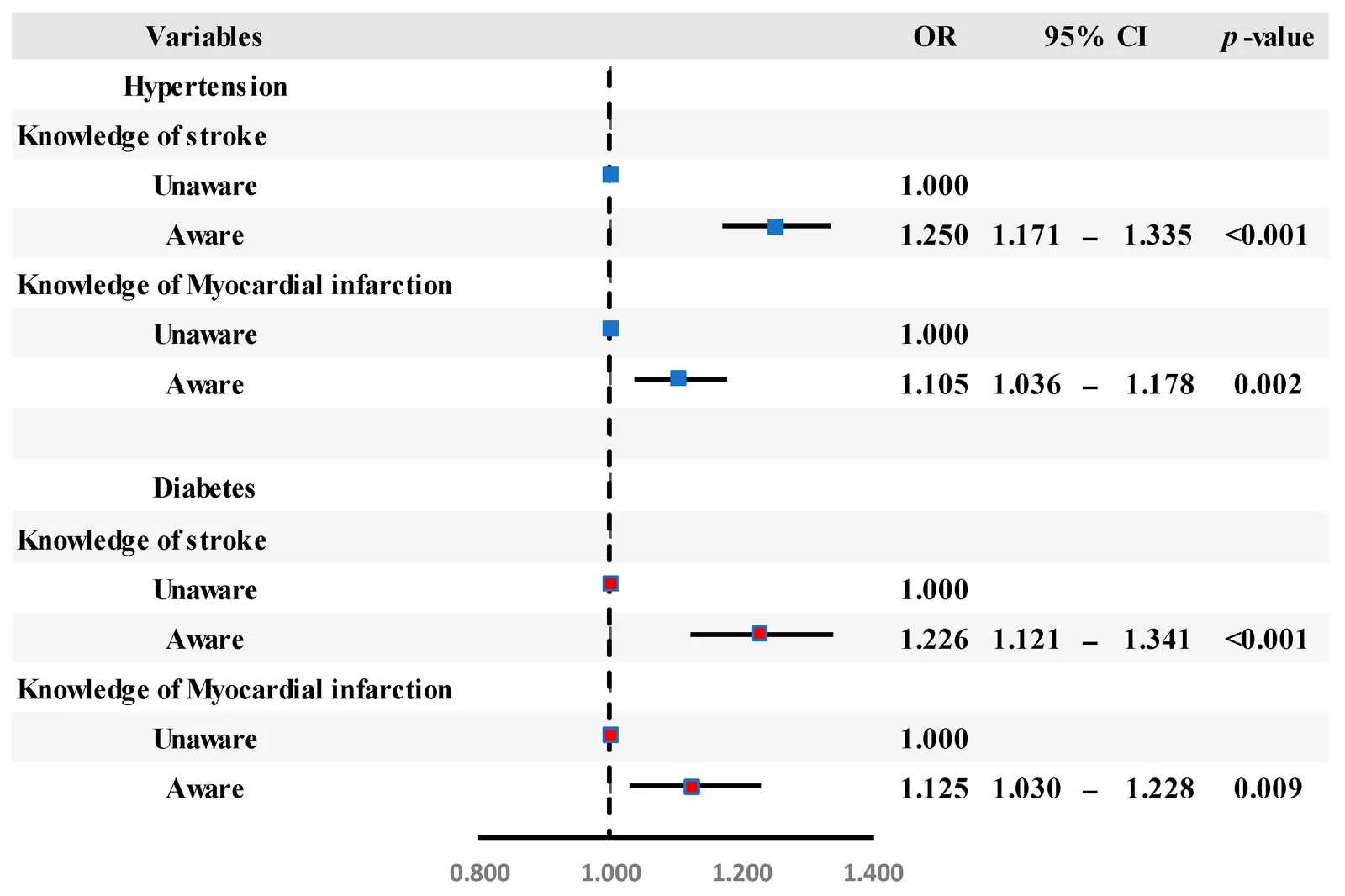
Forest plot illustrating the association between stroke and myocardial infarction knowledge and participation in cancer screening. Abbreviations: OR, odds ratio; CI, confidence interval. ORs and 95% CIs were derived from multivariable logistic regression models. The models were adjusted for sex, age, education level, marital status, residential area, employment status, average monthly household income, self-rated health status, smoking status, alcohol consumption, regular physical activity, obesity, depressive symptoms, and unmet healthcare needs. The “unaware” group was used as the reference category. Horizontal lines indicate 95% CIs. Blue squares represent hypertension and red squares represent diabetes. The vertical dashed line indicates an odds ratio of 1.0.

Figure 2 and Table S2 present the associations between knowledge of stroke and MI and participation in cancer screening among patients with chronic diseases. Among participants with hypertension, knowledge of stroke (OR: 1.250; 95% CI: 1.171–1.335; *p* < 0.001) and MI symptoms (OR: 1.105; 95% CI: 1.036–1.178; *p* = 0.002) was significantly associated with greater odds of undergoing cancer screening. Similarly, among patients with diabetes, those who were aware of stroke (OR: 1.226; 95% CI: 1.121–1.341; *p* < 0.001) and MI symptoms (OR: 1.125; 95% CI: 1.030–1.228; *p* = 0.009) were significantly more likely to undergo cancer screening than those who were unaware of the respective symptoms.

Furthermore, when knowledge scores were analyzed as continuous variables, a graded positive association was observed for stroke knowledge (Table S3). Each 1-point increase in the stroke knowledge score was significantly associated with a higher likelihood of undergoing cancer screening across all diagnostic groups. However, the association between MI knowledge score and cancer screening participation was positive but was not deemed significant (hypertension, OR: 1.023, *p* = 0.059; diabetes, OR: 1.028, *p* = 0.079). Moreover, subgroup analyses were performed according to comorbidity status (Table S4). For comparison, among individuals without hypertension or diabetes (*n* = 61,085), both knowledge of stroke (OR: 1.283; 95% CI: 1.212–1.357; *p* < 0.001) and MI (OR: 1.067; 95% CI: 1.008–1.128; *p* = 0.024) were significantly associated with increasing screening participation. Similarly, among individuals with both hypertension and diabetes (*n* = 13,308), knowledge of stroke was significantly associated with higher screening participation (OR: 1.191; 95% CI: 1.068–1.328; *p* = 0.002). Meanwhile, knowledge of myocardial infarction demonstrated a positive but non-significant association (OR: 1.092; 95% CI: 0.982–1.213; *p* = 0.103).

Furthermore, sensitivity analyses excluding the “unmet healthcare needs” variable demonstrated that the positive associations between knowledge of acute symptoms and cancer screening participation remained robust and significant across all diagnostic groups. These findings support the robustness of the primary findings and reduce concern regarding overadjustment bias (Table S5).

### 3.3. Association Between Knowledge of Stroke and Myocardial Infarction and Participation in Cancer Screening by Subgroups

**Figure 3:**
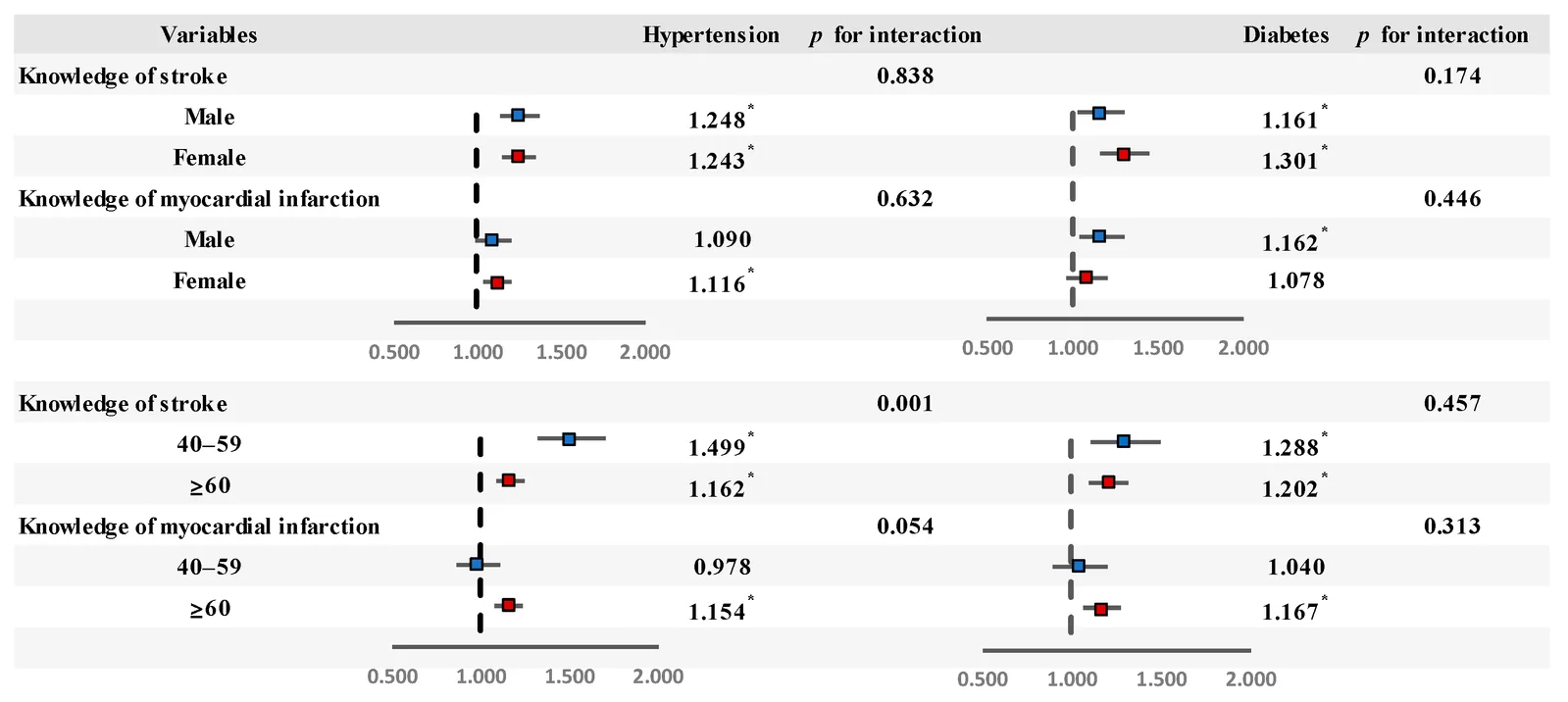
Forest plot of the association between knowledge of stroke and myocardial infarction and participation in cancer screening by subgroup. * Indicates significance (*p* < 0.05). Odds ratios (ORs) and 95% confidence intervals (CIs). All data points and horizontal error bars represent ORs and 95% CIs derived from multivariable logistic regression models. Separate models were conducted for individuals with hypertension (**left** panels) and diabetes (**right** panels), stratified by sex (**top** panels) and age group (**bottom** panels). Horizontal lines represent 95% CIs. Models were adjusted for education level, marital status, residential area, employment status, average monthly household income, self-rated health status, smoking status, alcohol consumption, regular physical activity, obesity, depressive symptoms, and unmet healthcare needs. Additionally, sex-stratified models were adjusted for age, and age-stratified models were adjusted for sex. The “unaware” group was used as the reference category. Formal interaction testing identified a significant interaction between stroke knowledge and age among patients with hypertension (*p* for interaction = 0.001). No significant interactions were observed for sex or the remaining age comparisons (*p* for interaction ≥ 0.05). Blue squares indicate males and the 40–59 age group, while red squares indicate females and the ≥60 age group. The vertical dashed line represents an odds ratio of 1.0.

Figure 3 (Table S6) presents the association between knowledge of stroke and MI symptoms and participation in cancer screening among patients with chronic diseases, stratified by age and sex. Among patients with hypertension, knowledge of stroke was significantly associated with higher odds of participation in cancer screening in both men (OR: 1.248; 95% CI: 1.133–1.376; *p* < 0.001) and women (OR: 1.243; 95% CI: 1.146–1.348; *p* < 0.001). Similarly, for knowledge of MI symptoms, a significant positive association was observed among women (OR: 1.116; 95% CI: 1.032–1.207; *p* = 0.006) but not among men (OR: 1.090; 95% CI: 0.992–1.198; *p* = 0.072).

Among patients with diabetes, knowledge of stroke symptoms was significantly associated with higher odds of participating in cancer screening in both women (OR: 1.301; 95% CI: 1.166–1.452; *p* < 0.001) and men (OR: 1.161; 95% CI: 1.032–1.307; *p* = 0.013). Similarly, for MI symptom knowledge, the association was significant among men (OR: 1.162; 95% CI: 1.037–1.302; *p* = 0.010), but not among women (OR: 1.078; 95% CI: 0.967–1.203; *p* = 0.177).

With regard to age groups, among individuals with hypertension, stroke symptom knowledge was associated with higher odds of participating in cancer screening in both those aged 40–59 years (OR: 1.499; 95% CI: 1.321–1.701; *p* < 0.001) and those aged ≥60 years (OR: 1.162; 95% CI: 1.084–1.245; *p* < 0.001). For MI symptom knowledge, a significant association was observed among those aged ≥60 years (OR: 1.154; 95% CI: 1.078–1.236; *p* < 0.001), whereas the estimate for those aged 40–59 years was not statistically significant (OR: 0.978; 95% CI: 0.866–1.105; *p* = 0.720). Among patients with diabetes, stroke symptom knowledge was similarly associated with higher odds of cancer screening participation in both those aged 40–59 years (OR: 1.288; 95% CI: 1.108–1.498; *p* = 0.001) and those aged ≥60 years (OR: 1.202; 95% CI: 1.096–1.319; *p* < 0.001). Likewise, for MI symptom knowledge, the association was significant among those aged ≥60 years (OR: 1.167; 95% CI: 1.066–1.278; *p* = 0.001), while the estimate for those aged 40–59 years was not (OR: 1.040; 95% CI: 0.899–1.204; *p* = 0.597).

To validate these subgroup differences, formal interaction tests were conducted within the multivariate models. A significant interaction was observed between stroke knowledge and age among patients with hypertension (*p* for interaction = 0.001), indicating a more pronounced association in the middle-aged group. However, no significant interactions were observed by sex in either disease group or for the remaining age comparisons (all *p* for interaction ≥ 0.05).

## 4. Discussion

### 4.1. Core Findings

This study demonstrated that knowledge of stroke and MI symptoms was significantly associated with cancer screening participation among patients with hypertension and diabetes. Stroke symptom knowledge was consistently associated with higher participation across all subgroups, with the most pronounced association observed among middle-aged individuals (40–59 years). By contrast, the association between knowledge of MI symptoms and cancer screening participation was more evident among older adults (≥60 years). Although no previous studies have directly examined this specific cross-disease association, the present findings are consistent with evidence linking limited knowledge of stroke and MI symptoms to lower participation in health checkups among adults [34]. They also align with studies demonstrating positive associations between general health knowledge and cancer prevention behaviors across various demographics [35,36,37]. Furthermore, these findings are consistent with previous studies identifying health knowledge as a key factor associated with preventive behaviors among patients with coronary artery disease [38,39], gastroesophageal reflux disease [40], and stroke [10]. Greater health knowledge may enhance understanding of personal health status and be associated with greater engagement in preventive health behaviors [41].

### 4.2. Mechanistic Interpretation

Interpretation of these findings requires careful consideration of the conceptual distinction between the independent and dependent variables. Recognizing acute symptoms of stroke or MI represents a reactive behavior primarily driven by the need to respond to an acute, life-threatening event [42]. By contrast, participation in cancer screening reflects a proactive preventive behavior aimed at detecting disease before symptom onset [43,44]. Therefore, the observed association should not be interpreted as evidence of a direct mechanistic pathway linking cardiovascular symptom knowledge to cancer screening. Instead, knowledge of these warning symptoms may serve as a proxy for broader health-related attributes. Individuals with greater symptom knowledge may possess higher overall health literacy, a more proactive preventive health orientation, and greater health awareness [10,45,46]. Even after adjusting for unmet medical needs as a proxy for healthcare accessibility, awareness of specific symptoms was associated with participation in screening. This finding could partly represent a broader preventive orientation, suggesting that the observed behavior may be consistent with more active, self-directed health management, rather than solely reflecting opportunistic screening during routine chronic disease care.

It is also noteworthy that while the dichotomized analysis (score 5 vs. 0–4) for MI knowledge demonstrated a significant positive association with cancer screening, the continuous score analysis did not. This mismatch may suggest a pattern similar to the threshold value. Specifically, partial knowledge of MI symptoms (e.g., scores of 1 to 4) may not capture the broader health awareness associated with participation in cancer screening, whereas comprehensive knowledge (a perfect score of 5) may characterize a subgroup with higher health awareness. On the other hand, the graded association observed for stroke knowledge may suggest that greater stroke awareness is associated with broader preventive health behaviors.

Regarding sex differences, while stratum-specific estimates for MI symptom knowledge reached statistical significance in certain subgroups, formal interaction tests by sex were not significant. Although exploring these tentative patterns may generate hypotheses for future research—such as whether men and women perceive or respond to cardiovascular health threats differently [47,48,49,50,51]—these subgroup variations should be interpreted strictly as exploratory pending longitudinal verification. Age-related differences, however, were supported by a significant interaction for stroke knowledge among individuals with hypertension. Among middle-aged individuals, the association between knowledge of stroke and participation in cancer screening was more pronounced. This pattern may reflect greater attention to disease prevention in middle age [52,53] and awareness that stroke can also occur during this life stage [54]. By contrast, among older adults, the association with knowledge of MI was more evident. Although MI and cancer share common systemic risk factors [55], the increased rate of cancer diagnosis observed after MI has been attributed primarily to intensified medical surveillance [56]. This increased clinical contact, coupled with heightened awareness of serious health threats, may be associated with greater participation in health screening.

### 4.3. Public Health Implications

Patients with hypertension and diabetes often perceive a higher cardiovascular disease risk [57], a perception associated with greater engagement in preventive health behaviors [58]. Our findings suggest that comprehensive health knowledge may be associated with broader engagement in preventive health behaviors. Health knowledge plays a crucial role in recognizing and responding to warning signs of preventable diseases, and timely recognition of stroke warning signs is associated with improved functional outcomes [59]. A multidimensional approach may be needed to improve health knowledge through public education, technology-based information dissemination, and community-based programs. Future studies could examine whether health education strategies that extend beyond information delivery are associated with broader preventive health behaviors, including cancer screening. Importantly, interaction testing identified a significant interaction only by age in the association between stroke knowledge and cancer screening participation among patients with hypertension. Consideration of both this confirmed interaction and the remaining exploratory findings may help inform tailored health education, although any targeted subgroup strategies remain exploratory pending longitudinal verification. Since no significant interactions by sex were observed, further research is needed to determine whether educational approaches should differ by sex.

### 4.4. Research Limitations

This study has some limitations that should be considered. First, the cross-sectional design of the CHS compared “current symptom knowledge” (assessed in 2023) with cancer screening performed within the past 2 years, precluding causal inferences. Specifically, reverse causality is possible, as recent contact with the preventive healthcare system for cancer screening may have improved patients’ overall health literacy, including awareness of stroke or MI symptoms, rather than symptom knowledge influencing screening behavior. Furthermore, as symptom knowledge and cancer screening history were assessed using self-reported survey data, the findings are subject to recall and social desirability bias.

Second, the dependent variable aggregated all types of cancer screening into a binary outcome (yes/no) within the preceding 2 years, reflecting the inherent structure of the CHS questionnaire. Consequently, the analysis lacks stratification by specific screening type, gender-specific screening items (e.g., breast or cervical cancer), or the distinction between organized national screening programs and opportunistic private health check-ups. Because sex- and age-specific screening indications could not be evaluated, it remains unclear whether participants adhered to the specific guidelines for individual cancer types. We acknowledge this measurement limitation, which should be considered when interpreting the clinical applicability of the present findings. Given this aggregation, the observed screening participation should be interpreted as a general indicator of preventive healthcare utilization rather than precise, guideline-concordant screening adherence.

Third, although multiple covariates were adjusted for, the potential for residual bias due to critical unmeasured confounders remains. Specifically, the dataset lacked information on personal or family history of cardiovascular disease or cancer, physician screening recommendation status, and validated health literacy measures. Furthermore, a single adjustment for household income cannot completely eliminate residual socioeconomic confounding. For instance, information regarding the type of medical coverage (National Health Insurance versus Medical Aid) was unavailable. In South Korea, Medical Aid beneficiaries represent a socioeconomically vulnerable population with unique patterns of and barriers to healthcare access. Finally, intrinsic health awareness could not be measured, meaning the observed associations may have been partially influenced by healthy user bias.

Fourth, as this study utilized publicly available secondary data, information on the annual frequency of outpatient visits was unavailable. Although unmet healthcare needs were included as a proxy for healthcare access, this measure reflects access barriers rather than the actual frequency of clinical contact, making it an imperfect surrogate for surveillance bias. Importantly, because of the cross-sectional design, temporal precedence among symptom knowledge, healthcare access barriers, and screening participation cannot be established. Consequently, conducting a formal mediation analysis to separate direct and indirect effects was not methodologically appropriate. However, to address potential overadjustment bias if this variable partially functioned as a mediator, we conducted sensitivity analyses excluding it (Table S5). The consistent results mitigate concerns that the primary associations were attributable to overadjustment. Future longitudinal studies linking CHS and NHIS claims data are warranted to evaluate the impact of healthcare utilization using precise measures of outpatient visit frequency.

Fifth, the exclusion of approximately 34.6% of the initial sample due to missing covariate data—predominantly household income—introduced potential attrition bias. We employed a complete-case analysis rather than multiple imputation, primarily because applying multiple imputation to complex, stratified, multistage survey data with specific sampling weights is methodologically challenging and can introduce its own unpredictable biases if the imputation model is misspecified. While our remaining sample size (*n* = 114,083) provided sufficient statistical power, we acknowledge that the missingness was not strictly random. As shown in Table S1, the excluded participants were disproportionately employed. Because employed adults in Korea frequently undergo mandatory workplace health checkups [60], excluding this economically active population likely altered the baseline prevalence of cancer screening in our sample. Consequently, we cannot fully quantify the potential deviation of our estimated odds ratios caused by this non-random missingness, which limits the generalizability of our findings to the broader working population.

## 5. Conclusions

Knowledge of MI and stroke symptoms was associated with cancer screening participation among patients with hypertension and diabetes in the Republic of Korea. Formal interaction testing indicated that the association for stroke knowledge was significantly more pronounced among middle-aged adults (40–59 years) with hypertension, while other subgroup patterns by sex and age were strictly exploratory pending longitudinal verification. These findings suggest that cardiovascular symptom knowledge may indicate broader preventive health engagement, including cancer screening among patients with hypertension and diabetes, and provide a basis for further research into comprehensive cardiovascular health education strategies.

## Figures and Tables

**Table 1:** Baseline characteristics of the study participants.

Variables	Hypertension					Diabetes				
	Total		Cancer Screening			Total		Cancer Screening		
			Yes					Yes		
	N	% *	N	% *	*p*-Value	N	% *	N	% *	*p*-Value
Total	45,949	100.0	31,958	70.4		20,357	100.0	14,216	70.4	
Knowledge of stroke					<0.001					<0.001
Unaware	18,563	39.0	11,929	36.0		8500	41.2	5530	38.3	
Aware	27,386	61.0	20,029	64.0		11,857	58.8	8686	61.7	
Knowledge of myocardial infarction					<0.001					<0.001
Unaware	22,548	48.7	14,850	46.2		10,009	49.3	6677	46.8	
Aware	23,401	51.3	17,108	53.8		10,348	50.7	7539	53.2	
Sex					0.002					0.597
Male	20,143	49.9	14,290	50.4		9982	54.0	7051	54.2	
Female	25,806	50.1	17,668	49.6		10,375	46.0	7165	45.8	
Age, years					<0.001					<0.001
40–49	2448	9.1	1679	8.9		1056	8.3	688	7.6	
50–59	6628	20.9	4876	22.1		2988	20.7	2125	21.1	
≥60	36,873	70.0	25,403	69.0		16,313	71.0	11,403	71.3	
Education level					<0.001					<0.001
Elementary school or lower	18,245	27.5	11,659	24.5		7755	27.7	5062	25.6	
Middle school graduate	7713	15.5	5563	15.6		3539	16.1	2526	16.1	
High school graduate	12,156	31.2	8788	32.1		5645	31.8	4064	32.2	
College graduate or higher	7835	25.8	5948	27.8		3418	24.4	2564	26.1	
Marital status					<0.001					<0.001
Married	30,487	70.7	22,712	75.1		13,737	70.2	10,217	74.2	
Divorce/widow	14,254	25.9	8612	22.4		6000	25.8	3672	22.8	
Single	1208	3.4	634	2.5		620	4.1	327	3.0	
Residential area					<0.001					0.037
Metropolitan	11,844	42.5	8229	42.6		5426	42.4	3806	42.9	
Urban	11,378	35.3	8072	36.0		5231	35.7	3652	35.7	
Rural	22,727	22.1	15,657	21.4		9700	21.9	6758	21.4	
Employment status					<0.001					<0.001
Yes	22,812	49.6	14,819	46.8		9698	47.1	7331	50.7	
No	23,137	50.4	17,139	53.2		10,659	52.9	6885	49.3	
Average monthly household income					<0.001					<0.001
<100	11,755	17.0	6959	14.3		4984	17.4	3001	15.0	
100–199	11,734	21.3	8083	20.0		5346	22.4	3702	21.5	
200–299	8324	18.0	6063	18.1		3924	18.8	2818	18.5	
300–399	3613	10.2	2692	10.6		1593	9.5	1202	10.0	
≥400	10,523	33.6	8161	37.0		4510	31.8	3493	34.9	
Self-rated health status					<0.001					<0.001
Above average	29,482	69.4	21,541	72.4		11,625	61.3	8586	64.8	
Poor	16,467	30.6	10,417	27.6		8732	38.7	5630	35.2	
Smoking					0.243					0.087
Yes	17,921	44.3	12,476	44.1		9153	49.6	6363	49.1	
No	28,028	55.7	19,482	55.9		11,204	50.4	7853	50.9	
Drinking					<0.001					<0.001
Yes	34,415	80.5	24,439	81.8		15,443	80.5	10,950	81.5	
No	11,534	19.5	7519	18.2		4914	19.5	3266	18.5	
Regular physical activity					<0.001					<0.001
Yes	7647	16.7	5781	18.2		3297	16.0	2506	17.3	
No	38,302	83.3	26,177	81.8		17,060	84.0	11,710	82.7	
Obesity					<0.001					0.005
Yes	18,310	43.1	13,030	43.9		7875	40.9	5505	40.9	
No	27,639	56.9	18,928	56.1		12,482	59.1	8711	59.1	
Depressive symptom					<0.001					<0.001
Yes	2344	5.4	1315	4.5		1241	6.5	705	5.4	
No	43,605	94.6	30,643	95.5		19,116	93.5	13,511	94.6	
Unmet healthcare needs					<0.001					<0.001
Yes	2397	5.0	1429	4.2		1015	4.8	557	3.7	
No	43,552	95.0	30,529	95.8		19,342	95.2	13,659	96.3	

* Weighted.

## Data Availability

The data supporting the findings of this study are available from the KDCA under public-use conditions for researchers who submit an approved research proposal. The dataset is publicly available through the Korea CHS at https://chs.kdca.go.kr/chs/index.do. It was accessed for this study on 24 November 2024.

## Supplementary Materials

The following supporting information can be downloaded at https://www.mdpi.com/article/10.3390/healthcare14172782/s1. Table S1: Comparison of baseline sociodemographic characteristics between included and excluded participants; Table S2: Association between knowledge of stroke and myocardial infarction and participation in cancer screening; Table S3: Association between continuous knowledge scores and participation in cancer screening; Table S4: Association between knowledge of stroke and myocardial infarction and participation in cancer screening according to comorbidity status; Table S5: Sensitivity analysis of the association between knowledge of stroke and myocardial infarction and cancer screening participation after excluding unmet healthcare needs; Table S6: Association between knowledge of stroke and myocardial infarction and participation in cancer screening by subgroup.

## Abbreviations

The following abbreviations are used in this manuscript:

BMI	Body Mass Index
CHS	Community Health Survey
CI	Confidence interval
KDCA	Korea Disease Control and Prevention Agency
MI	Myocardial infarction
NCSP	National Cancer Screening Program
NHIS	National Health Insurance Service
OR	Odds ratio
VIF	Variance inflation factor
WHO	World Health Organization
