# Supplementary material for: Association of Stroke and Myocardial Infarction Symptom Knowledge with Cancer Screening Participation Among Patients with Hypertension and Diabetes: A Cross-Sectional Study in Korea
Source: Healthcare (Basel). 2026 Sep 1;14(17):2782. doi: 10.3390/healthcare14172782 (PMC13566089; doi:10.3390/healthcare14172782)
Supplement: Supplementary file 1 [file healthcare-14-02782-s001.zip › healthcare-4469342-supplementary.pdf]

Supplementary Materials

Table S1. Comparison of baseline sociodemographic characteristics between included and excluded participants

Variables	Patients with hypertension/diabetes (*n* = 52,998)		Individuals without hypertension/diabetes (*n* = 61,085)		Excluded participants (*n* = 71,790)		*p*-value
	N	%	N	%	N	%	
Sex					(Missing value *n* = 0)		< 0.001
Male	23,828	45.0	25,240	41.3	34,211	47.7	
Female	29,170	55.0	35,845	58.7	37,579	52.4	
Age, years					(Missing value *n* = 0)		< 0.001
40–49	3,048	5.8	16,327	26.7	14,441	20.1	
50–59	7,988	15.1	17,253	28.2	17,512	24.4	
≥60	41,962	79.2	27,505	45.0	39,837	55.5	
Education level					(Missing value *n* = 100)		< 0.001
Elementary school or lower	20,394	38.5	10,285	16.8	16,167	22.6	
Middle school graduate	8,883	16.8	6,595	10.8	9,401	13.1	
High school graduate	14,348	27.1	20,884	34.2	24,047	33.5	
College graduate or higher	9,373	17.7	23,321	38.2	22,075	30.8	
Marital status					(Missing value *n* = 41)		< 0.001
Married	35,589	67.2	46,546	76.2	54,392	75.8	
Divorce/widow	15,935	30.1	10,822	17.7	13,087	18.2	
Single	1,474	2.8	3,717	6.1	4,270	6.0	
Residential area					(Missing value *n* = 0)		< 0.001
Metropolitan	13,873	26.2	18,667	30.6	14,730	20.5	
Urban	13,286	25.1	18,674	30.6	18,076	25.2	
Rural	25,839	48.8	23,744	38.9	38,984	54.3	
Employment status					(Missing value *n* = 7)		< 0.001
Yes	26,393	49.8	21,046	34.5	48,124	67.0	
No	26,605	50.2	40,039	65.5	23,659	33.0	
Average monthly household income					(Missing value *n* = 52,994)		< 0.001
<100	13,103	24.7	7,199	11.8	4,476	23.8	
100–199	13,388	25.3	9,569	15.7	4,005	21.3	
200–299	9,721	18.3	9,872	16.2	3,113	16.6	
300–399	4,266	8.0	7,522	12.3	1,824	9.7	
≥400	12,520	23.6	26,923	44.1	5,378	28.6	
Self-rated health status					(Missing value *n* = 1)		< 0.001

Above average	34,076	64.3	51,613	84.5	57,702	80.4	
Poor	18,922	35.7	9,472	15.5	14,087	19.6	
Smoking					(Missing value ***n*** = 7)		< 0.001
Yes	21,337	40.3	22,633	37.1	30,108	41.9	
No	31,661	59.7	38,452	62.9	41,675	58.1	
Drinking					(Missing value ***n*** = 6)		< 0.001
Yes	39,963	75.4	50,971	83.4	58,459	81.4	
No	13,035	24.6	10,114	16.6	13,325	18.6	
Regular physical activity					(Missing value ***n*** = 21)		< 0.001
Yes	8,924	16.8	13,011	21.3	16,890	23.5	
No	44,074	83.2	48,074	78.7	54,879	76.5	
Obesity					(Missing value ***n*** = 2,642)		< 0.001
Yes	20,449	38.6	15,026	24.6	22,260	32.2	
No	32,549	61.4	46,059	75.4	46,888	67.8	
Depressive symptom					(Missing value ***n*** = 550)		< 0.001
Yes	2,711	5.1	2,010	3.3	2,083	2.9	
No	50,287	94.9	59,075	96.7	69,157	97.1	
Unmet healthcare needs					(Missing value ***n*** = 11,467)		0.355
Yes	2,743	5.2	3,580	5.9	3,326	5.5	
No	50,255	94.8	57,505	94.1	56,997	94.5	

Table S2. Association between knowledge of stroke and myocardial infarction and participation in cancer screening

Variables	Cancer screening										
	Hypertension					Diabetes					
	OR	95% CI			*p*-value	OR	95% CI			*p*-value	
Knowledge of stroke											
Unaware	1.000					1.000					
Aware	1.250	1.171	–	1.335	< 0.001	1.226	1.121	–	1.341	< 0.001	
Knowledge of myocardial infarction											
Unaware	1.000					1.000					
Aware	1.105	1.036	–	1.178	0.002	1.125	1.030	–	1.228	0.009	
Sex											
Male	1.000					1.000					
Female	1.138	1.040	–	1.247	0.005	1.234	1.091	–	1.397	0.001	
Age, years											
40–49	0.544	0.478	–	0.618	< 0.001	0.482	0.407	–	0.571	< 0.001	
50–59	0.769	0.702	–	0.842	< 0.001	0.695	0.616	–	0.784	< 0.001	
≥60	1.000					1.000					
Education level											
Elementary school or lower	0.701	0.635	–	0.775	< 0.001	0.752	0.660	–	0.857	< 0.001	
Middle school graduate	0.852	0.770	–	0.943	0.002	0.842	0.734	–	0.966	0.014	
High school graduate	0.873	0.802	–	0.951	0.002	0.865	0.772	–	0.968	0.012	
College graduate or higher	1.000					1.000					
Marital status											
Married	1.000					1.000					
Divorce/widow	0.672	0.629	–	0.717	< 0.001	0.682	0.624	–	0.746	< 0.001	
Single	0.506	0.438	–	0.585	< 0.001	0.602	0.493	–	0.734	< 0.001	
Residential area											
Metropolitan	1.000					1.000					
Urban	1.047	0.981	–	1.118	0.166	0.947	0.868	–	1.034	0.224	
Rural	0.982	0.921	–	1.047	0.573	0.901	0.821	–	0.988	0.027	
Employment status											
Yes	1.000					1.000					
No	0.728	0.686	–	0.773	< 0.001	0.676	0.621	–	0.737	< 0.001	
Average monthly household income											
<100	0.699	0.631	–	0.774	< 0.001	0.724	0.632	–	0.831	< 0.001	
100–199	0.730	0.665	–	0.801	< 0.001	0.768	0.678	–	0.870	< 0.001	
200–299	0.796	0.724	–	0.876	< 0.001	0.735	0.647	–	0.835	< 0.001	
300-399	0.855	0.765	–	0.956	0.006	0.868	0.740	–	1.019	0.084	
≥400	1.000					1.000					
Self-rated health status											
Above average	1.000					1.000					
Poor	0.872	0.821	–	0.927	< 0.001	0.795	0.734	–	0.862	< 0.001	
Smoking											
Yes	0.843	0.772	–	0.921	0.000	0.907	0.805	–	1.023	0.112	
No	1.000					1.000					
Drinking											
Yes	1.174	1.098	–	1.254	< 0.001	1.148	1.048	–	1.258	0.003	

No	1.000					1.000				
Regular physical activity										
Yes	1.000					1.000				
No	0.798	0.736	–	0.864	< 0.001	0.831	0.746	–	0.926	0.001
Obesity										
Yes	1.068	1.011	–	1.128	0.020	1.003	0.931	–	1.080	0.945
No	1.000					1.000				
Depressive symptom										
Yes	0.871	0.782	–	0.969	0.012	0.875	0.761	–	1.006	0.0613
No	1.000					1.000				
Unmet healthcare needs										
Yes	0.689	0.613	–	0.775	< 0.001	0.582	0.496	–	0.681	< 0.001
No	1.000					1.000				

Table S3. Association between continuous knowledge scores and participation in cancer screening

Variables	Cancer screening									
	Hypertension					Diabetes				
	OR	95% CI			*p*-value	OR	95% CI			*p*-value
Knowledge of stroke (per 1-point increase)	1.087	1.061	–	1.113	< 0.001	1.077	1.043	–	1.111	< 0.001
Knowledge of Myocardial infarction (per 1-point increase)	1.023	0.999	–	1.047	0.059	1.028	0.997	–	1.061	0.079
Sex										
Male	1.000					1.000				
Female	1.127	1.029	–	1.234	0.010	1.220	1.077	–	1.382	0.002
Age, years										
40–49	0.547	0.481	–	0.622	< 0.001	0.482	0.407	–	0.572	< 0.001
50–59	0.772	0.705	–	0.846	< 0.001	0.694	0.615	–	0.783	< 0.001
≥60	1.000					1.000				
Education level										
Elementary school or lower	0.709	0.642	–	0.784	< 0.001	0.759	0.665	–	0.866	< 0.001
Middle school graduate	0.852	0.770	–	0.944	0.002	0.844	0.736	–	0.969	0.016
High school graduate	0.871	0.800	–	0.948	0.001	0.864	0.772	–	0.968	0.011
College graduate or higher	1.000					1.000				
Marital status										
Married	1.000					1.000				
Divorce/widow	0.676	0.634	–	0.722	< 0.001	0.687	0.629	–	0.752	< 0.001
Single	0.503	0.435	–	0.582	< 0.001	0.598	0.490	–	0.729	< 0.001
Residential area										
Metropolitan	1.000					1.000				
Urban	1.048	0.981	–	1.119	0.162	0.951	0.871	–	1.038	0.258
Rural	0.984	0.923	–	1.049	0.626	0.904	0.824	–	0.992	0.032
Employment status										
Yes	1.000					1.000				
No	0.730	0.688	–	0.775	< 0.001	0.676	0.620	–	0.736	< 0.001

Average monthly household income										
<100	0.700	0.632	–	0.775	< 0.001	0.726	0.633	–	0.833	< 0.001
100–199	0.728	0.664	–	0.799	< 0.001	0.768	0.678	–	0.871	< 0.001
200–299	0.798	0.725	–	0.878	< 0.001	0.736	0.648	–	0.836	< 0.001
300–399	0.852	0.762	–	0.953	0.005	0.860	0.733	–	1.010	0.066
≥400	1.000					1.000				
Self-rated health status										
Above average	1.000					1.000				
Poor	0.870	0.819	–	0.925	< 0.001	0.792	0.731	–	0.859	< 0.001
Smoking										
Yes	0.839	0.768	–	0.916	< 0.001	0.901	0.799	–	1.016	0.089
No	1.000					1.000				
Drinking										
Yes	1.165	1.090	–	1.244	< 0.001	1.147	1.047	–	1.256	0.003
No	1.000					1.000				
Regular physical activity										
Yes	1.000					1.000				
No	0.797	0.736	–	0.863	< 0.001	0.830	0.745	–	0.925	0.001
Obesity										
Yes	1.064	1.007	–	1.124	0.027	1.001	0.930	–	1.078	0.973
No	1.000					1.000				
Depressive symptom										
Yes	0.865	0.777	–	0.963	0.008	0.870	0.756	–	1.001	0.051
No	1.000					1.000				
Unmet healthcare needs										
Yes	0.685	0.61	–	0.77	< 0.001	0.579	0.494	–	0.678	< 0.001
No	1.000					1.000				

Table S4. Association between knowledge of stroke and myocardial infarction and participation in cancer screening according to comorbidity status

Variables	Cancer screening									
	Individuals without Hypertension/Diabetes (n = 61,085)					Both (n = 13,308)				
	OR	95% CI			p-value	OR	95% CI			p-value
Knowledge of stroke										
Unaware	1.000					1.000				
Aware	1.283	1.212	–	1.357	< 0.001	1.191	1.068	–	1.328	0.002
Knowledge of myocardial infarction										
Unaware	1.000					1.000				
Aware	1.067	1.008	–	1.128	0.024	1.092	0.982	–	1.213	0.103
Sex										
Male	1.000					1.000				
Female	1.381	1.290	–	1.477	< 0.001	1.194	1.031	–	1.382	0.018
Age, years										
40–49	0.588	0.545	–	0.634	< 0.001	0.482	0.377	–	0.615	< 0.001
50–59	0.768	0.717	–	0.823	< 0.001	0.702	0.602	–	0.820	< 0.001
≥60	1.000					1.000				
Education level										
Elementary school or lower	0.708	0.647	–	0.774	< 0.001	0.848	0.723	–	0.995	0.043
Middle school graduate	0.897	0.817	–	0.985	0.023	0.912	0.774	–	1.074	0.269
High school graduate	0.812	0.766	–	0.860	< 0.001	0.939	0.814	–	1.084	0.390
College graduate or higher	1.000					1.000				
Marital status										
Married	1.000					1.000				
Divorce/widow	0.737	0.690	–	0.786	< 0.001	0.619	0.559	–	0.686	< 0.001
Single	0.501	0.458	–	0.547	< 0.001	0.627	0.489	–	0.804	0.000
Residential area										
Metropolitan	1.000					1.000				
Urban	1.058	1.001	–	1.118	0.045	0.963	0.870	–	1.066	0.469
Rural	0.910	0.859	–	0.965	0.002	0.911	0.819	–	1.014	0.087
Employment status										
Yes	1.000					1.000				
No	0.781	0.740	–	0.825	< 0.001	0.645	0.581	–	0.715	< 0.001

Average monthly household income										
<100	0.546	0.495	–	0.603	< 0.001	0.782	0.668	–	0.914	0.002
100–199	0.656	0.603	–	0.712	< 0.001	0.808	0.700	–	0.933	0.004
200–299	0.714	0.661	–	0.771	< 0.001	0.766	0.661	–	0.887	0.000
300–399	0.776	0.719	–	0.839	< 0.001	0.860	0.703	–	1.052	0.143
≥400	1.000					1.000				
Self-rated health status										
Above average	1.000					1.000				
Poor	0.969	0.906	–	1.036	0.359	0.789	0.718	–	0.868	< 0.001
Smoking										
Yes	0.863	0.806	–	0.923	< 0.001	0.850	0.740	–	0.978	0.023
No	1.000					1.000				
Drinking										
Yes	1.158	1.082	–	1.238	< 0.001	1.177	1.054	–	1.313	0.004
No	1.000					1.000				
Regular physical activity										
Yes	1.000					1.000				
No	0.878	0.828	–	0.930	< 0.001	0.762	0.667	–	0.869	< 0.001
Obesity										
Yes	0.964	0.914	–	1.016	0.174	1.049	0.962	–	1.142	0.278
No	1.000					1.000				
Depressive symptom										
Yes	0.836	0.741	–	0.945	0.004	1.001	0.854	–	1.173	0.9896
No	1.000					1.000				
Unmet healthcare needs										
Yes	0.682	0.624	–	0.746	< 0.001	0.589	0.492	–	0.705	< 0.001
No	1.000					1.000				

Table S5. Sensitivity analysis of the association between knowledge of stroke and myocardial infarction and cancer screening participation after excluding unmet healthcare needs

Variables	Cancer screening									
	Hypertension					Diabetes				
	OR	95% CI			*p*-value	OR	95% CI			*p*-value
Knowledge of stroke										
Unaware	1.000					1.000				
Aware	1.087	1.061	–	1.113	< 0.001	1.079	1.045	–	1.113	< 0.001
Knowledge of myocardial infarction										
Unaware	1.000					1.000				
Aware	1.023	0.999	–	1.047	0.059	1.027	0.995	–	1.059	0.094
Sex										
Male	1.000					1.000				
Female	1.117	1.020	–	1.224	0.018	1.204	1.063	–	1.363	0.003
Age, years										
40–49	0.545	0.479	–	0.619	< 0.001	0.473	0.399	–	0.560	< 0.001
50–59	0.768	0.701	–	0.841	< 0.001	0.687	0.609	–	0.774	< 0.001
≥ 60	1.000					1.000				
Education level										
Elementary school or lower	0.711	0.644	–	0.786	< 0.001	0.758	0.665	–	0.864	< 0.001
Middle school graduate	0.853	0.770	–	0.944	0.002	0.844	0.736	–	0.967	0.015
High school graduate	0.870	0.799	–	0.947	0.001	0.864	0.772	–	0.967	0.011
College graduate or higher	1.000					1.000				
Marital status										
Married	1.000					1.000				
Divorce/widow	0.674	0.632	–	0.719	< 0.001	0.684	0.626	–	0.747	< 0.001
Single	0.502	0.434	–	0.580	< 0.001	0.590	0.484	–	0.718	< 0.001
Residential area										
Metropolitan	1.000					1.000				
Urban	1.045	0.979	–	1.116	0.183	0.947	0.868	–	1.033	0.217
Rural	0.981	0.921	–	1.046	0.563	0.900	0.820	–	0.987	0.026
Employment status										
Yes	1.000					1.000				
No	0.735	0.693	–	0.781	< 0.001	0.681	0.625	–	0.741	< 0.001
Average monthly household income										
<100	0.692	0.625	–	0.765	< 0.001	0.716	0.625	–	0.821	< 0.001
100–199	0.726	0.662	–	0.797	< 0.001	0.764	0.674	–	0.865	< 0.001
200–299	0.797	0.724	–	0.877	< 0.001	0.735	0.647	–	0.835	< 0.001
300–399	0.853	0.763	–	0.954	0.005	0.865	0.737	–	1.014	0.074
≥400	1.000					1.000				

Self-rated health status										
Above average	1.000					1.000				
Poor	0.859	0.808	–	0.913	< 0.001	0.784	0.723	–	0.849	< 0.001
Smoking										
Yes	0.835	0.764	–	0.912	< 0.001	0.894	0.793	–	1.008	0.068
No	1.000					1.000				
Drinking										
Yes	1.164	1.089	–	1.243	< 0.001	1.145	1.046	–	1.254	0.003
No	1.000					1.000				
Regular physical activity										
Yes	1.000					1.000				
No	0.799	0.737	–	0.866	< 0.001	0.840	0.754	–	0.936	0.002
Obesity										
Yes	1.065	1.008	–	1.125	0.026	1.007	0.935	–	1.084	0.864
No	1.000					1.000				
Depressive symptom										
Yes	0.828	0.744	–	0.921	0.001	0.815	0.711	–	0.935	0.004
No	1.000					1.000				

Table S6. Association between knowledge of stroke and myocardial infarction and participation in cancer screening by subgroup

Variables	Cancer Screening								
	Hypertension								
	Male				Female				
	OR	95% CI			OR	95% CI			*p* for interaction
Knowledge of stroke									
Unaware	1.000				1.000				
Aware	1.248	1.133	–	1.376	1.243	1.146	–	1.348	0.838
Knowledge of myocardial infarction									
Unaware	1.000				1.000				
Aware	1.090	0.992	–	1.198	1.116	1.032	–	1.207	0.632

Variables	Diabetes								
	Male				Female				
	OR	95% CI			OR	95% CI			*p* for interaction
Knowledge of stroke									
Unaware	1.000				1.000				
Aware	1.161	1.032	–	1.307	1.301	1.166	–	1.452	0.174
Knowledge of myocardial infarction									
Unaware	1.000				1.000				
Aware	1.162	1.037	–	1.302	1.078	0.967	–	1.203	0.446

Variables	Hypertension								
	40–59 years				≥ 60 years				
	OR	95% CI			OR	95% CI			*p* for interaction
Knowledge of stroke									
Unaware	1.000				1.000				
Aware	1.499	1.321	–	1.701	1.162	1.084	–	1.245	0.001
Knowledge of myocardial infarction									
Unaware	1.000				1.000				
Aware	0.978	0.866	–	1.105	1.154	1.078	–	1.236	0.054

Variables	Diabetes								
	40–59 years				≥ 60 years				
	OR	95% CI			OR	95% CI			*p* for interaction
Knowledge of stroke									
Unaware	1.000				1.000				
Aware	1.288	1.108	–	1.498	1.202	1.096	–	1.319	0.457
Knowledge of myocardial infarction									
Unaware	1.000				1.000				
Aware	1.040	0.899	–	1.204	1.167	1.066	–	1.278	0.313
